# Purity and Uncertainty Study of CRM Betulin by DSC

**DOI:** 10.1007/s13659-020-00261-1

**Published:** 2020-08-18

**Authors:** Li Zhang, Peng-Hui Yuan, De-Zhi Yang, Yan-Cai Bi, Bin Su, Bao-Xi Zhang, Fu-Qing Wang, Yang Lu, Guan-Hua Du

**Affiliations:** 1 Beijing City Key Laboratory of Polymorphic Drugs, Center of Pharmaceutical Polymorphs, Institute of Materia Medica, Chinese Academy of Medical Sciences and Peking Union Medical College, Beijing, 100050 People’s Republic of China; 2 Soteria Pharmaceutical Co., Ltd., Laiwu, 271100 People’s Republic of China; 3 China Pharmaceutical Enterprises Association, Beijing, 100044 People’s Republic of China; 4 Beijing City Key Laboratory of Drug Target and Screening Research, National Center for Pharmaceutical Screening, Institute of Materia Medica, Chinese Academy of Medical Sciences and Peking Union Medical College, Beijing, 100050 People’s Republic of China

**Keywords:** Betulin, DSC, Purity determination, Uncertainty study

## Abstract

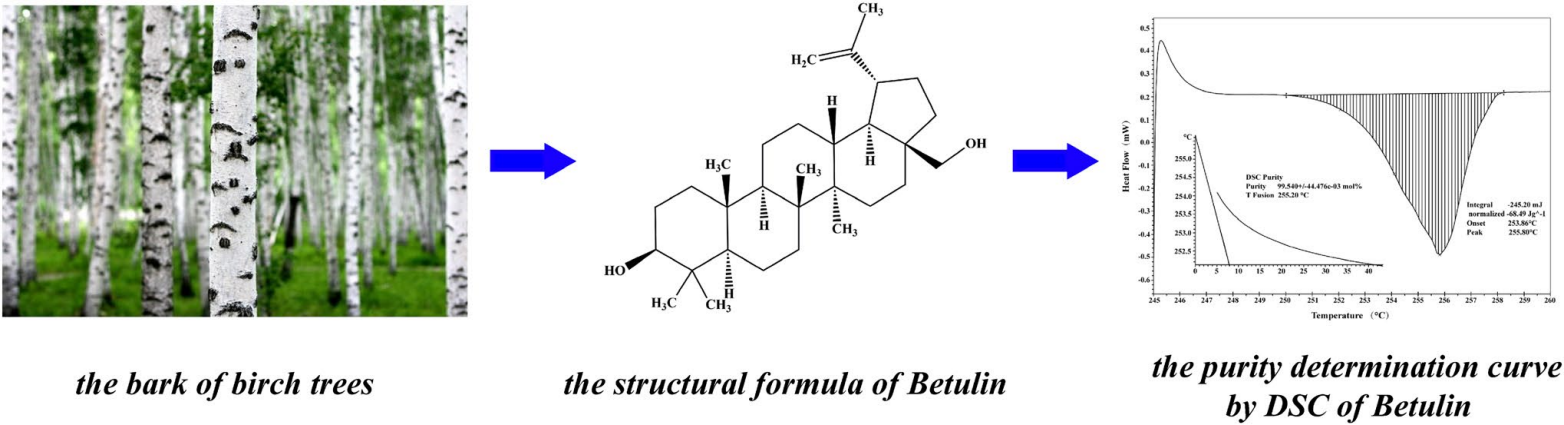

## Introduction

**Fig. 1:**
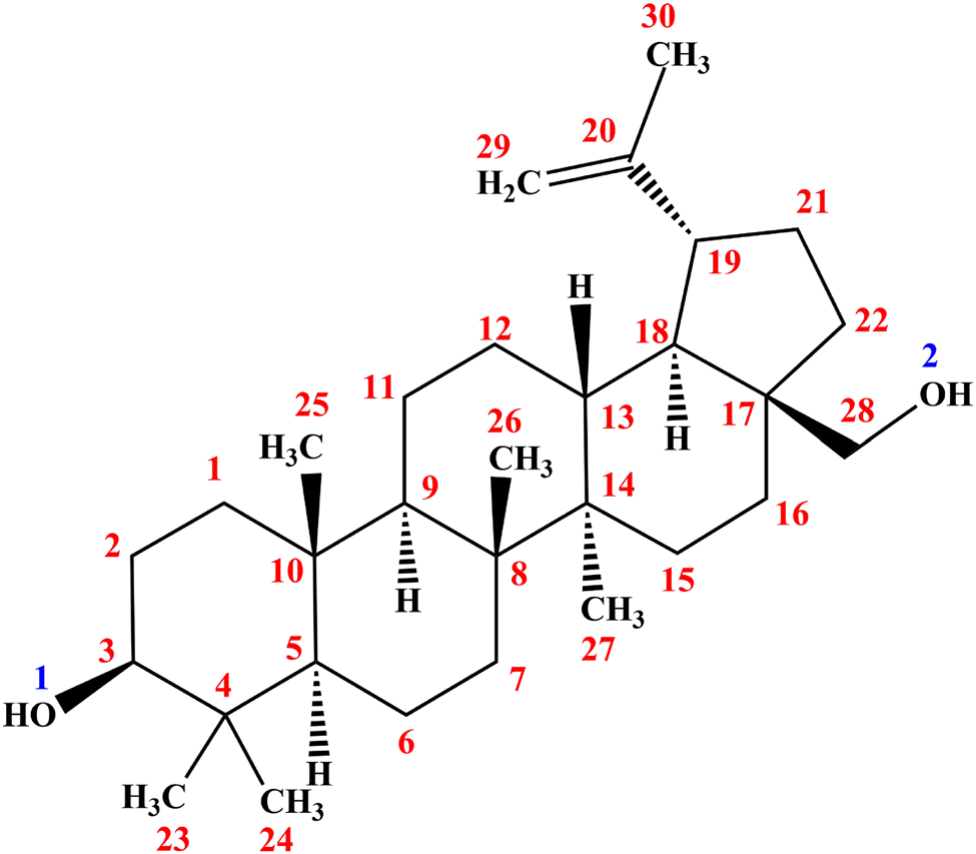
Structural formula of Betulin

Betulin [BE; betulinol, 3β-lup-20(29)-en-3,28-diol] belongs to a class of pentacyclic triterpenes and is the predominant compound of the outer bark of birch tree (*Betula*) [1]. Figure 1 shows the structural formula of BE. BE can be obtained from more than 200 plants species, although the richest source of BE are those belonging to Betulaceae family, especially *Betula alba*, *B. pubescens*, *B. platyphylla*, and *B. pendula *[2]. Pharmacological investigations have revealed that BE has many notable biological properties, such as anti-bacterial, anti-inflammatory, anti-fungal, and anti-viral effects, that work effectively on lesions; it also acts as a prophylactic and curative agent [3]. BE exhibits antitumor activity against many types of tumor cell lines: cervix carcinoma HeLa cells, hepatoma HepG2 cells, lung adenocarcinoma A549 cells, breast cancer MCF-7 cells, human gastric carcinoma, human pancreatic carcinoma, and different melanoma and skin cancer cell lines [4, 5]. As a promising clinical medication, BE has received increasing attention over the past few decades. Hence, the establishment of an accurate quantitative analysis method for BE is essential to enhance its utilization.

Certified reference materials (CRMs) are used for qualitative and quantitative analyses and have extremely important applications in the food and pharmaceutical fields [6]. In addition to accuracy and precision, which are the classical aims of analytical chemistry, resolving uncertainty is a recent and modern goal of CRMs [7, 8]. Therefore, not only the purity of a CRM but also its uncertainty value must be known. The concept of uncertainty can reveal the quantitative interplay of the individual working steps of a method and thus lead to a deep understanding of its critical points.

Traditionally, chromatographic techniques, such as high-performance liquid chromatography (HPLC), in conjunction with other analytical techniques have been used to determine the purity of CRMs. The purity of BE can be determined by HPLC [9–12]. Recently, differential scanning calorimetry (DSC) has been used to quantitatively determine the purity of compounds that are at least 98% [13].

This paper describes the development process of the CRM of BE, including the preparation process of the material, evaluation of the homogeneity and stability of samples within and between groups, and employment of a strategy to assign reference values for the CRM. Following ISO Guide 34 and 35 [14, 15] the procedures included qualitative and quantitative analyses, verification, the study of homogeneity and stability, purity determination, and uncertainty evaluation. Additionally, in the current article, the CRM of BE was investigated and described by DSC for the first time.

## Results and Discussion

### Investigation of Heating Rate

The present study investigated three different heating rates (0.3, 0.5, and 0.7 K/min) for purity measurement. The purity values under three different heating rates were all 99.55%. Results showed that the purity value during the measurements was stable and had good parallelism when the heating rate was between 0.3–0.7 K/min. Therefore, the final experimental conditions were determined at heating rate of 0.5 K/min and heating interval of 245–260 °C. Table 1 lists the measurement data from the investigation of heating rate.

**Table 1:** Measurement data from the investigation of sample weighing of Betulin

No.	Sample weight (mg)	Endothermic peak area (Mj)
1	1.97	138.98
2	2.21	154.68
3	2.81	198.36
4	3.27	225.11
5	3.68	251.13
6	4.12	281.93

### Investigation of Sample Weighing

**Fig. 2:**
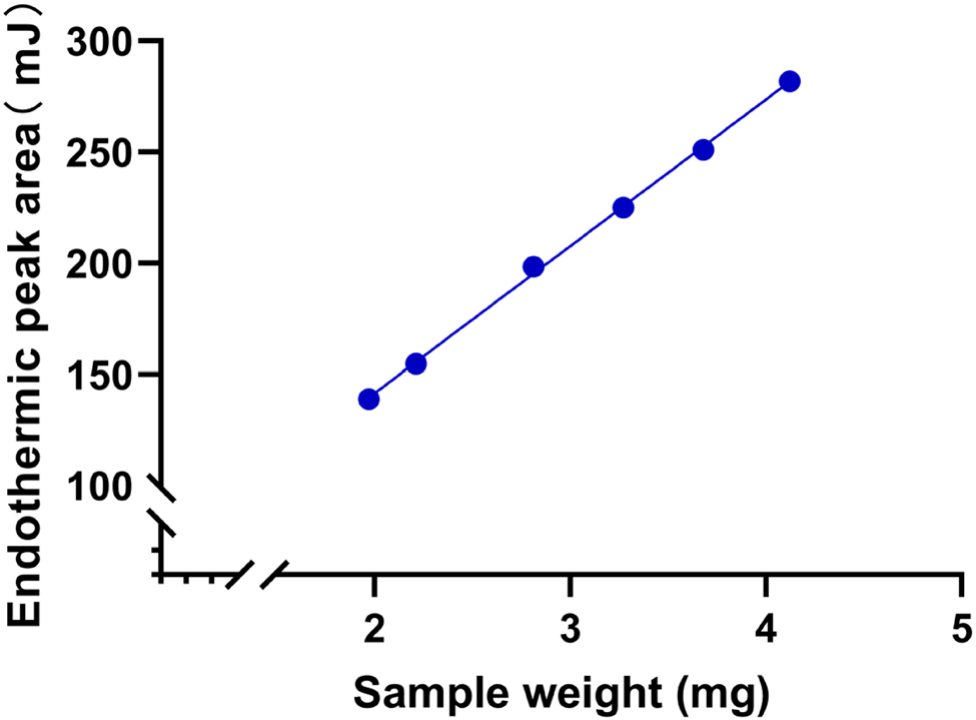
Linear regression result of the investigation of sample weighing of Betulin

A standard curve was drawn by using the sample mass as the x-coordinate and the endothermic peak area as the y-coordinate. Within the mass range of 1.97–4.12 mg, the regression linear equation was Y = 66.0284X + 9.6196, and the correlation coefficient *R*^2^ was 0.9991 (n = 6). The results indicated a good linear relationship between the sample mass and endothermic peak area. Consequently, DSC can be used for determining the purity of the CRM of BE. Figure 2 shows the linear regression result of the investigation of sample weighing.

### Homogeneity Test

The means of the squares between groups (MS_between_) and within each group (MS_within_) were 6.99 × 10^–9^ and 4.47 × 10^–9^, respectively. The calculated F-value was 1.46, which was lower than the critical F-value (1.74). No significant differences were found in the purity of BE during the homogeneity test. Table 2 provides the measurement data from the homogeneity tests.

**Table 2:** Measurement data from the homogeneity test of Betulin

No.	Relative purity(*Y*_*ij*_) (%)			$\overline{{Y}_{j}}$(%)
	Samp1	Samp2	Samp3	
1	99.54	99.54	99.55	99.54
2	99.53	99.54	99.54	99.53
3	99.54	99.55	99.55	99.54
4	99.53	99.54	99.53	99.53
5	99.54	99.55	99.55	99.54
6	99.53	99.54	99.55	99.54
7	99.54	99.53	99.53	99.53
8	99.54	99.54	99.53	99.53
9	99.53	99.53	99.54	99.53
10	99.54	99.54	99.55	99.54
11	99.55	99.55	99.54	99.54
12	99.53	99.55	99.54	99.54
13	99.54	99.53	99.53	99.53
14	99.55	99.54	99.54	99.54
15	99.54	99.54	99.55	99.54
16	99.53	99.54	99.55	99.54
17	99.54	99.54	99.54	99.54
18	99.55	99.53	99.52	99.53
19	99.54	99.55	99.54	99.54
20	99.54	99.54	99.55	99.54
21	99.53	99.53	99.54	99.53
22	99.53	99.54	99.53	99.53
23	99.54	99.54	99.55	99.54
24	99.53	99.54	99.54	99.53
25	99.54	99.55	99.54	99.54
Average				99.54
SD				4.83 × 10^–5^

The analytical data were assessed by one-way analysis of variance.

### Stability Test

**Fig. 3:**
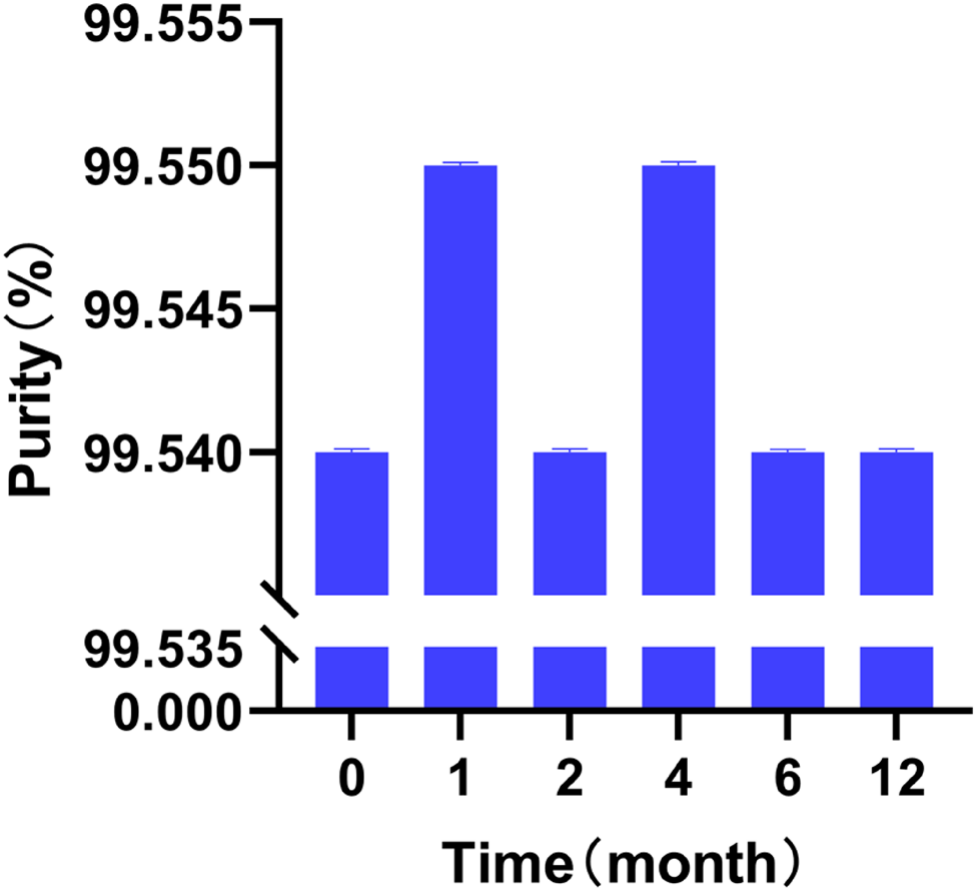
Results of long-term stability test of Betulin

The data for the short-term stability tests were examined by Student’s t-test, and those for the long-term stability test were assessed by regression analysis. The statistical analysis showed that the CRM of BE can be stored at 25 °C for at least 12 months. Table 3 lists the short-term stability test measurement data. Figure 3 exhibits the results of long-term stability test of BE.

**Table 3:** Measurement data from the short-term stability test of Betulin

Condition	Time (day)	Purity (%)									Mean (%)	SD
		Vials 1			Vials 2			Vials 3				
		1	2	3	1	2	3	1	2	3		
60 °C	0	99.53	99.54	99.55	99.54	99.55	99.54	99.53	99.54	99.53	99.54	0.000078
	5	99.54	99.53	99.54	99.53	99.54	99.55	99.54	99.55	99.55	99.54	0.000078
	10	99.55	99.55	99.56	99.53	99.56	99.56	99.55	99.53	99.54	99.55	0.000120
	15	99.55	99.53	99.54	99.54	99.55	99.54	99.54	99.53	99.53	99.54	0.000078
	20	99.54	99.55	99.55	99.54	99.55	99.54	99.53	99.54	99.56	99.54	0.000088
Strong light (4500 l× ± 500 l×)	0	99.54	99.55	99.53	99.55	99.54	99.55	99.54	99.55	99.55	99.54	0.000073
	5	99.55	99.54	99.54	99.53	99.54	99.55	99.55	99.54	99.56	99.54	0.000088
	10	99.55	99.53	99.55	99.53	99.53	99.55	99.55	99.54	99.55	99.54	0.000097
	15	99.54	99.54	99.54	99.55	99.56	99.54	99.56	99.56	99.54	99.55	0.000097
	20	99.55	99.54	99.55	99.54	99.54	99.55	99.54	99.54	99.55	99.54	0.000053

### Minimum Sampling Weight Test

In this part, DSC was used to investigate the minimum sampling weight of the CRM of BE. In the experiment, four different weights, namely, 1.0, 2.0, 3.0, and 4.0 mg, were used to study the minimum sampling weight. Six parallel experiments were conducted for each mass, and the results are shown in Table 4.

**Table 4:** Result of the minimum sampling amount test

No.	Weight (mg)	Purity (%)	No.
0.94	99.09	99.18	0.0026857
1.09	98.79		
1.06	99.29		
1.10	99.61		
1.16	99.11		
1.10	99.19		
2.30	99.56	99.56	0.0001163
1.91	99.55		
2.06	99.56		
2.12	99.55		
1.96	99.58		
1.96	99.57		
2.96	99.53	99.55	0.0001001
3.02	99.55		
3.17	99.55		
3.08	99.56		
3.00	99.56		
3.14	99.55		
4.02	99.56	99.55	0.0000775
3.90	99.55		
3.99	99.55		
3.88	99.56		
3.98	99.54		
3.91	99.55		

The results by calculations were as follows: $t_{10}^{0.05}$(1.0, 2.0) = 3.475, $t_{10}^{0.05}$(2.0, 3.0) = 1.803, $t_{10}^{0.05}$(2.0, 4.0) = 1.698, and $t_{10}^{0.05}$(3.0, 4.0) = 0.360. The results of t-test showed that the minimum sample quantity of “betulin purity standard material” was 2.0 mg.

### Purity Determination

**Fig. 4:**
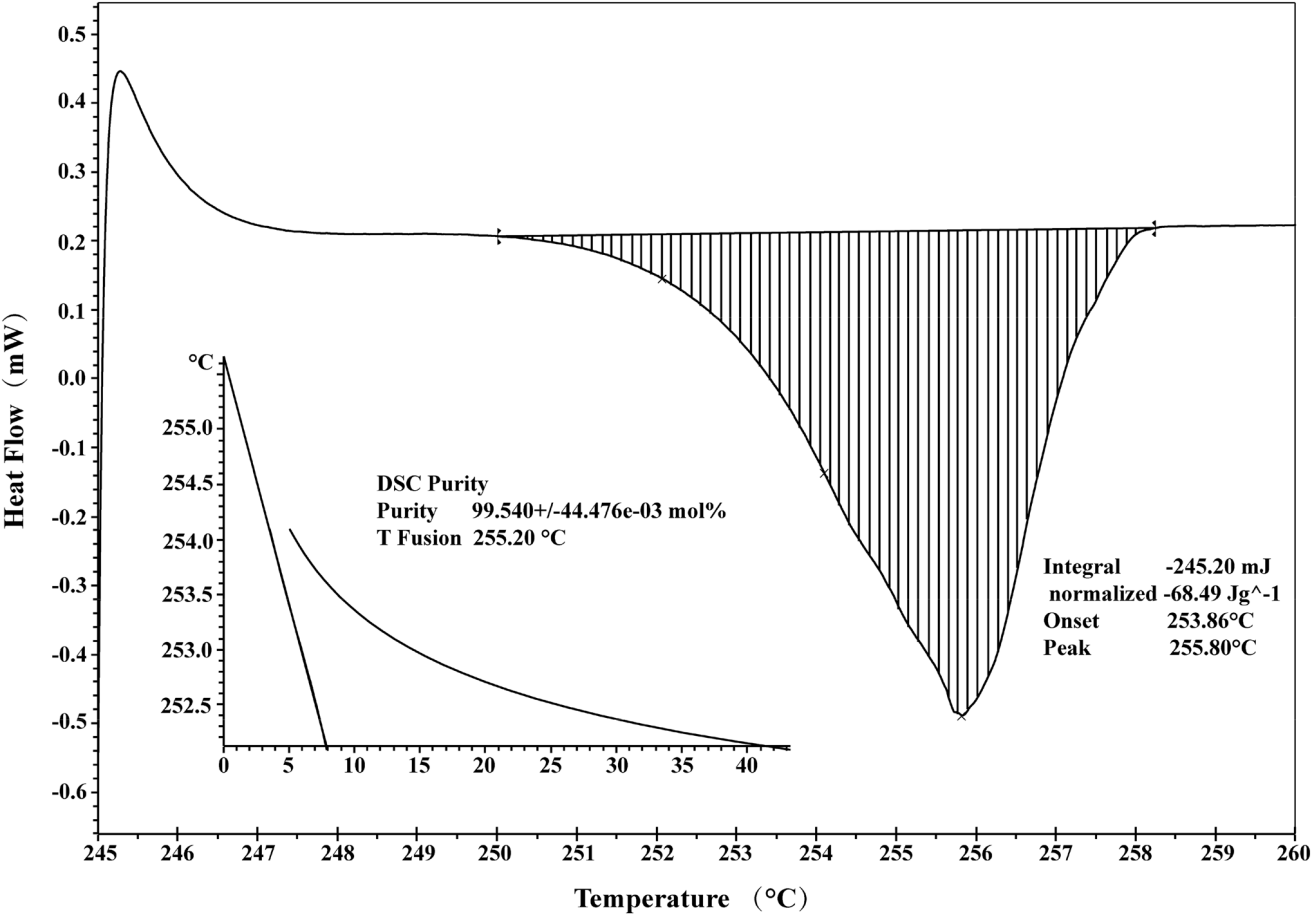
The DSC curve of Betulin

Figure 4 illustrates the DSC curve of BE and the endothermic melting peak at 256.5 °C. The purity determination by DSC was based on Van’t Hoff’s law [16]. The purity of BE was calculated in accordance with the following formula:

1 $$x_{DSC} = (1 - \frac{{(T_{0} - T_{m} )\Delta H}}{{RT_{0}^{2} }}) \times 100\% = (1 - \frac{{QMF(T_{0} - T_{m} )}}{{mRT_{0}^{2} }}) \times 100\%$$

where *x*_DSC_ is the purity, and *Q* and *m* are the thermal values calibrated by stannum and sample mass, respectively. *M* is the molar mass of BE. T_0_ is the melting temperature of the pure compound; T_m_ is the actual melting temperature; R is gas constant; ΔH is the molar enthalpy of melting, and y is the mole fraction of impurity in the substance.

### Value Characterization

The purity of the analyte was calculated, and the results of purity determination are listed in Table 5. The mean purity was 99.56% with a standard deviation of 1.906%.

**Table 5:** Purity of analyte as determined by the DSC method

No.	Weight (mg)	Purity (%)
1	3.58	99.54
2	3.54	99.55
3	3.35	99.57
4	3.25	99.59
5	3.43	99.56
6	3.34	99.56
7	3.48	99.54
8	3.54	99.58
9	3.33	99.53
10	3.59	99.55

### Uncertainty Evaluation

The factors that affect the accuracy of the results in a measurement include environmental conditions, operational conditions, accuracy in weighing, the instrument used for measurement, molar melting enthalpy analysis, and accuracy of the analytical software. The analysis indicated that the uncertainty of measurement was derived from the following factors: the uncertainty in measurements of the melting enthalpy of stannum, measurement error, weighing error, temperature error, and calculation error of the DSC analysis software.

#### Uncertainty from Melting Enthalpy Measurements of Stannum (u_B1(DSC)_)

Table 6 lists the melting enthalpy of stannum (*u*_B1(DSC)_) measured by DSC.

**Table 6:** Melting enthalpy of Stannum measured by the DSC method

No.	Melting enthalpy (J/g)	SD
1	− 59.70	0.0004448
2	− 59.70	0.0004127
3	− 59.70	0.0004184
4	− 59.69	0.0003722
5	− 59.72	0.0004161
6	− 59.69	0.0004044
7	− 59.70	0.0004250
8	− 59.70	0.0003955
9	− 59.70	0.0004078
10	− 59.71	0.0003906

The uncertainty of *u*_B1(DSC)_ was calculated using the following expression:

$$u_{{B1\left( {DSC} \right)}} = \frac{s}{{\sqrt n \times \overline{\Delta H} }} = 4.64 \times 10^{ - 5} .$$

#### Uncertainty from Measurement Error (u _A1(DSC)_)

The *u *_A1(DSC)_ was calculated using the following expression:

$$u_{{\text{A1(DSC)}}} = \frac{s}{{\sqrt n \times \overline{P} }} = {6}.{03} \times 10^{{ - {5}}}$$

#### Uncertainty from Weighing Error (u _B2(DSC)_)

The *u*_B2(DSC)_ was calculated using the following expression:

$$u_{B2(DSC)} = \frac{{\left( {\frac{\partial X}{{\partial m}}dm} \right)}}{{k \times m_{\min } }}= - 8.575 \times 10^{ - 5}$$

#### Uncertainty from Temperature Error (u _B3(DSC)_)

The *u *_B3(DSC)_ was calculated using the following expression:

$$u_{B3(DSC)} = {{\left( {\frac{\partial X}{{\partial T_{0} }} + \frac{\partial X}{{\partial T_{m} }}} \right)} \mathord{\left/ {\vphantom {{\left( {\frac{\partial X}{{\partial T_{0} }} + \frac{\partial X}{{\partial T_{m} }}} \right)} k}} \right. \kern-\nulldelimiterspace} k} = 1.719 \times 10^{ - 4}$$

#### Uncertainty from Systematic Deviation of the DSC Analysis Software (u _B4(DSC)_)

Table 6 shows the systematic deviation of the DSC analysis software from the purity determination.

The *u*_B4(DSC)_ was estimated using the following expression:

$$u_{{B4\left( {DSC} \right)}} = \frac{{d_{\max } }}{{k \times \overline{P} }} = \, 2.579 \times 10^{ - 4}.$$

#### Combined Uncertainty of CRM

**Fig. 5:**
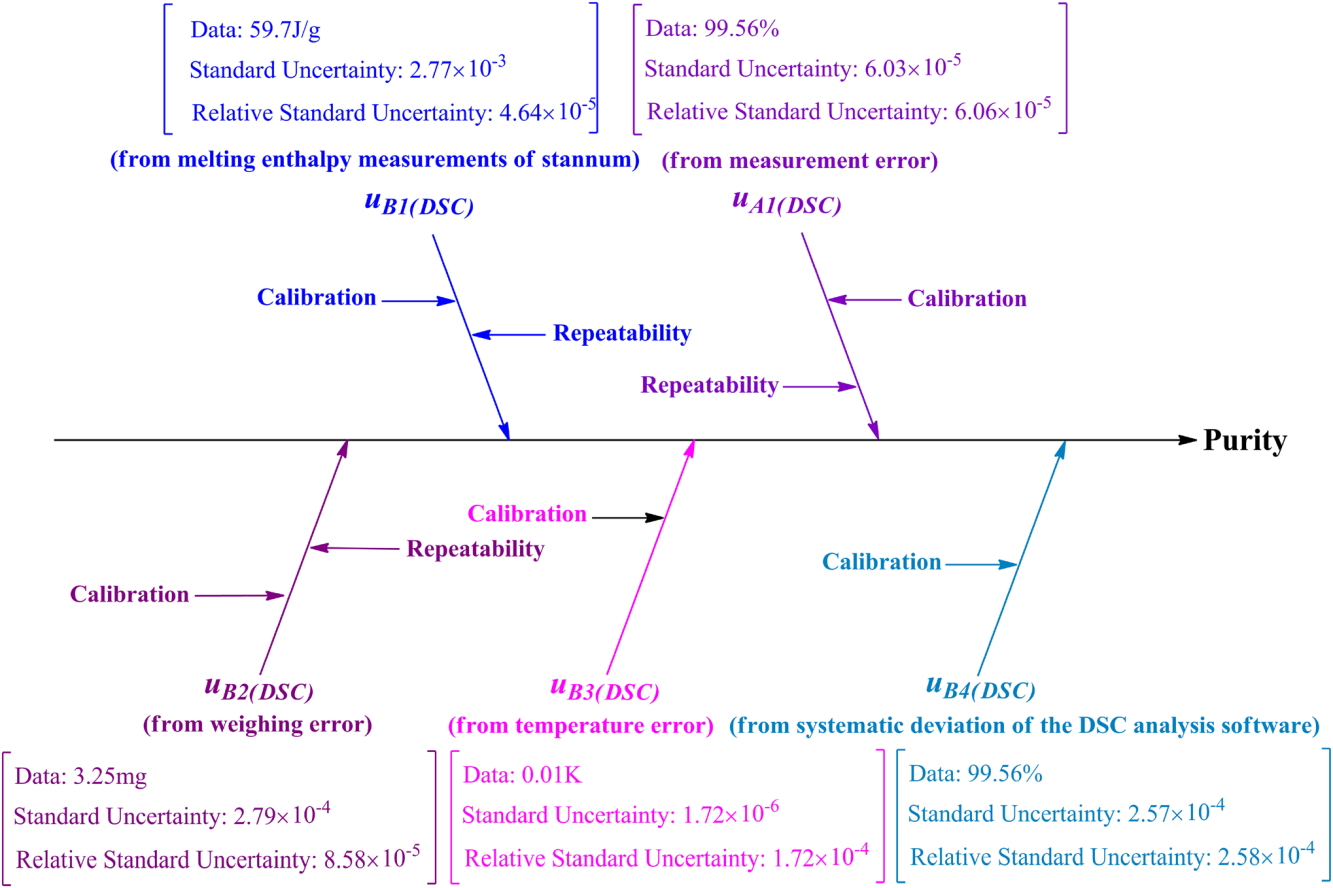
Cause and effect diagram showing the possible sources of uncertainty in the DSC method

The uncertainty of CRM arising from *u*_B1(DSC)_, *u*_A1(DSC)_, *u*_B2(DSC)_, *u*_B3(DSC)_, and *u*_B4(DSC)_ were calculated and discussed. Figure 5 shows the cause-and-effect diagram revealing the possible sources of uncertainty in DSC.

The combined uncertainty of CRM was calculated using the following expression:

$$\begin{aligned} \frac{{u_{(DSC)} }}{{P_{(DSC)} }} = & \sqrt {u_{B1(DSC)}^{2} + u_{A1(DSC)}^{2} + u_{B2(DSC)}^{2} + u_{B3(DSC)}^{2} + u_{B4(DSC)}^{2} } = 3.305 \times 10^{ - 4} \\ u_{(DSC)} = & P_{(DSC)} \times \sqrt {u_{B1(DSC)}^{2} + u_{A1(DSC)}^{2} + u_{B2(DSC)}^{2} + u_{B3(DSC)}^{2} + u_{B4(DSC)}^{2} } \\ = & 99.56\% \times 3.305 \times 10^{ - 4} = 3.290 \times 10^{ - 4} \\ \end{aligned}$$

#### Expanded Uncertainty (Uc) of CRM

The *U*c of CRM was calculated using a coverage factor of 2, which is associated with a confidence level of approximately 95%.

$$Uc(x) = k_{p} u_{(DSC)} = 0.07\%.$$

### Results of CRM Analysis

The purity of the CRM of BE was estimated at 99.56% ± 0.07% (k = 2) within a typical 95% confidence interval based on the combined value assignments and the *U*c by DSC.

### Method Validation and Value Certification

The purity of BE was certified by the mass balance method, which obtained a value of 99.54% ± 0.24% (k = 2) within a typical 95% confidence interval. The t-test indicated no significant difference between the results of the two methods. The certified value was expressed as the mean of the purity obtained by the two methods. The certified value of the CRM of BE was 99.6% ± 0.5% (k = 2) within a typical 95% confidence interval.

## Conclusion

In this study, a reliable, sensitive, and rapid DSC method was developed for the purity determination of a new CRM of BE. The purity of BE was determined by DSC method and certified by the mass balance method. The results of the two methods were consistent, indicating that DSC method can be used as an alternative technique for the purity determination of materials. The validation data presented here indicate that DSC method can be used to determine the purity of BE with consistent results. Compared with the traditional mass balance method, DSC method is faster and provides higher accuracy and precision. Furthermore, this method has good reproducibility and requires no corresponding reference standard and sample preparation. DSC method can be used as an alternative reference method to provide accurate data. The results can be compared with those of the routine methods employed for testing BE. We envision that DSC method can be used extensively in tests and evaluation processes in the pharmaceutical industry.

The new CRM of BE can be used for the calibration of analytical instruments, assessment of test methods, and securing the accuracy and comparability of measurement data. The new CRM of BE has been approved by the national administrative committee for CRM (GBW09593). This reference material can also be widely used to control the quality of BE-related pharmaceutical and foods formulations.

## Experiment Section

### Materials

BE was obtained from Yuancheng Co., Ltd. (Wu Han, China). All other reagents were of analytical grade.

### Preparation of Reference Material

BE (50 g) was dissolved in 95% ethanol at an external temperature of 60 °C in a round-bottom flask, and then kept at 60 °C until the sample had dissolved. Then the solution was subjected to hot filtering and retained at 4 °C overnight. After the crystals had completely precipitated, they were filtered and vacuum dried to obtain highly pure crystals of BE, which were dried at 60 °C for 4 h. The powder was milled and then passed through a 74-μm sieve. The fraction was homogenized in a rotating mixer prior to filling. Samples of 50 mg of the homogenized powder were dispensed into each dark glass vial, which were then sealed. Six hundred bottles were prepared for each batch of BE. The samples were stored at 25 °C for studies of homogeneity and stability.

### Determination of Homogeneity and Stability

Studies of homogeneity and stability were performed using the DSC method. The between-vial homogeneity was determined by analyzing one replicate in each of the 25 randomly selected bottles from each batch. The within-vial homogeneity was tested in three replicates for each vial. The results were statistically evaluated.

Eighteen samples were selected for the short-term stability study. The samples were divided into six different groups, each group containing three samples. The six groups of the sample were respectively stored at 60 °C and under light conditions (4500 l× ± 500 l×) for 0, 1, and 2 weeks. Another 36 samples were selected for the long-term stability study. The samples were divided into six different groups, each group containing six samples. The six groups of the sample were stored at 25 °C for 0, 1, 2, 4, 6, and 12 months under lighted condition, respectively.

### Characterization

The DSC analysis of the samples was conducted using a DSC1 system (Mettler-Toledo Inc, Switzerland). The samples (at approximately 3–5 mg) were hermetically sealed in aluminum crucibles to prevent any mass loss in the presence of moisture. All sample measurements were performed in triplicate at a heating rate of 0.5 K/min and temperatures ranged from 245 to 260 °C with furnace gas N_2_. The purity results obtained by DSC are reported as the average of the triplicate determinations.
